# Risk factors and long-term outcome for postoperative intra-abdominal infection after hepatectomy for hepatocellular carcinoma

**DOI:** 10.1097/MD.0000000000006795

**Published:** 2017-04-28

**Authors:** Haowen Tang, Wenping Lu, Zhanyu Yang, Kai Jiang, Yongliang Chen, Shichun Lu, Jiahong Dong

**Affiliations:** a Institute of Hepatobiliary Surgery, Chinese PLA General Hospital; b Center for Hepatopancreatobiliary Diseases, Beijing Tsinghua Changgung Hospital, Tsinghua University Medical Center, Changping, Beijing, China.

**Keywords:** hepatectomy, hepatocellular carcinoma, long-term outcome, postoperative intra-abdominal infection, risk factor

## Abstract

Intra-abdominal infection (IAI) after hepatectomy is an important morbidity. Identification of risk factors that could be avoided in the perioperative period may reduce the prevalence of IAI after hepatectomy for hepatocelluar carcinoma (HCC).

Between January 1995 and December 2009, all patients with HCC who underwent curative liver resection were evaluated retrospectively. Long-term outcomes were compared in IAI patients and non-IAI patients after hepatectomy. Preoperative, intraoperative, and tumor-related factors that could be independent factors for postoperative IAI were identified.

Of 622 patients with HCC, 24 patients (3.9%) had IAI after hepatectomy. Both median survival and survival of patients with postoperative IAI were shorter than those for non-IAI patients (*P* < .05). Upon univariate analysis followed by multivariate analyses, three independent predictors for IAI were identified: weight loss (odds ratio [OR], 3.27; 95% confidence interval [CI], 1.17–9.11; *P* = .024), liver cirrhosis (0.28, 0.12–0.67, .004), and operative time >300 minutes (3.44, 1.46–8.12, .005).

IAI after hepatectomy affects outcome adversely. Preoperative weight loss, liver cirrhosis, and operative time >300 minutes are independent predictors of postoperative IAI.

## 1 Introduction

Hepatocellular carcinoma (HCC) is the fifth most common malignancy worldwide, with >500,000 new cases annually.^[1,2]^ Globally, half of these cases are estimated to occur in China because of the prevalence of infection by the hepatitis-B virus (HBV).^[2,3]^ Liver resection remains first-line treatment for HCC, with 5-year survival of around 40% to 50% in patients with good liver function reserve.^[4–10]^

Despite advances in surgical methods and perioperative management, the relatively high morbidity after hepatectomy for HCC (≈40%) is problematic.^[11]^ Specifically, intra-abdominal infection (IAI), with an incidence of approximately 9%, remains a commonly encountered and severe type of complication.^[12]^ Postoperative IAI has been reported to be associated with poor long-term outcomes for various types of cancer: colorectal cancer, colorectal liver metastasis, and lung cancer.^[13–15]^ The mechanism of action for postoperative IAI in promotion of colorectal cancer recurrence has been elucidated in mice and in vitro.^[13,16,17]^ However, with respect to HCC, the impact of postoperative IAI on long-term outcome has not been published widely, and few studies have revealed the risk factors of IAI after hepatectomy.

The primary goal of our study was to investigate the impact of postoperative IAI on long-term outcome in patients undergoing hepatectomy for HCC. Also, we aimed to clarify the predictors for postoperative IAI using multivariate analyses based on a large cohort of patients.

## 2 Methods

### 2.1 Patients

Between January 1995 and December 2009, the records of all patients with HCC who underwent curative liver resection at the Institute of Hepatobiliary Surgery, Chinese PLA General Hospital (Beijing, China) were evaluated retrospectively. The study protocol was approved by the Ethics Committees of PLA General Hospital. Written informed consent for treatment and use of patient data for clinical research was obtained from each patient before surgery.

### 2.2 Diagnosis and definitions

All patients were diagnosed as having HCC by pathology. Before liver resection, enhanced computed tomography or magnetic resonance imaging was undertaken for all patients. Preoperative imaging examinations were used to accurately diagnose the tumor number, tumor diameter, thrombi in the portal vein, or bile duct tumor thrombi (BDTT), which were confirmed by postoperative pathology. In the most general sense, IAI referred to infection in the pelvis, subdiaphragmatic spaces, or any other localized collection within the abdomen (with or [rarely] without involvement of the overlying peritoneum) with clinical signs: pyrexia >38 °C, pus from discharging cavities, aspirates of intra-abdominal collections or upon exploration of the abdomen, increased levels of inflammatory markers (white cell count), positive fluid, or blood culture.^[15,18–24]^ IAI presented as generalized peritonitis or as a localized abscess, and necessitates treatment with antibiotics or surgical intervention. Preoperative portal hypertension (PHT) was evaluated retrospectively: direct measurement of venous pressure was not undertaken routinely in our series. PHT was defined indirectly according to criteria set by the Barcelona clinic liver cancer (BCLC) as: esophageal varices detectable by endoscopy or platelet count <100,000/μL in association with splenomegaly (major diameter, >12 cm).^[25,26]^ Preoperative weight loss denoted a medical problem or condition when at least 5% of a person's body weight had been lost in the preceding month or 10% in the 6 months.^[27,28]^ Duration of follow-up was defined as the period between the surgical procedure and death or final observation of patients.

### 2.3 Indications for liver resection

Clinical indications for liver resection were: liver function (Child–Pugh criteria) of A or B; absence of severe pulmonary/cardiovascular diseases; liver-volume remnant in patients with normal liver parenchyma and liver cirrhosis of >30% and >50%, respectively.^[29]^

### 2.4 Surgical procedures

Surgery was undertaken via a right subcostal incision with a “J” extension. The liver was mobilized using standard procedures. Surgical ultrasonography was done to determine the plane for parenchymal transection. For multiple HCCs, anatomic resection denoted the complete removal of all tumor-bearing segments. An intermittent Pringle maneuver was utilized in cycles of clamping (15 minutes) and unclamping (5 minutes). Transection of liver parenchyma was done using an ultrasonic aspirator (CUSA; ValleyLab, Boulder, CO) or by the clamp-crushing method. Haemostasis on the raw surface of the liver was maintained using an argon beam coagulator (ValleyLab). Routine closed-suction abdominal drainage was employed.

### 2.5 Statistical analyses

Statistical analyses were carried out using SPSS v14.0 (IBM, Armonk, NY). Categorical variables were presented as number (percentage); the χ^2^ test or Fisher exact test was used for comparison, as appropriate. Continuous variables were expressed as mean with standard deviation (SD) or median with range; the analysis of variance was chosen. Survival curves and survival were calculated by the Kaplan–Meier method, and examined using the log-rank test. Univariate logistic regression analysis of risk factors was done to calculate the odds ratio (OR), and variables with *P* < .05 were subjected consecutively to multivariate logistic regression analysis to identify the independent predictors for postoperative IAI. A two-tailed *P* < .05 was considered significant.

## 3 Results

### 3.1 Patient characteristics

During the study period 622 patients with HCC who underwent curative liver resection at the Institute of Hepatobiliary Surgery, Chinese PLA General Hospital (Beijing, China) were enrolled. Parts of patients’ characteristics were listed below. Among the 622 patients analyzed, there were 545 men patients and 77 women; the median age was 50 years (range 11–80) and mean age was 50 years (SD, 11). Twenty-four patients (3.9%) had IAI after hepatectomy, and all were cured with antibiotics or by surgical intervention. Most prevalent causes of underlying liver disease were infection by the HBV (77%) or hepatitis C virus (2%). Seventy-eight percent (483/622) of patients had liver cirrhosis; 14% (89/622) had multiple tumors; 33% (205/622) had PHT; 27% (170/622) had increased levels of total bilirubin; 4% (22/622) had BDTT. Moreover, 2%, 81%, 10%, 8%, and 0% belonged to BCLC stages 0, A, B, C, and D, respectively.

### 3.2 Influence of postoperative IAI on long-term survival

**Figure 1:**
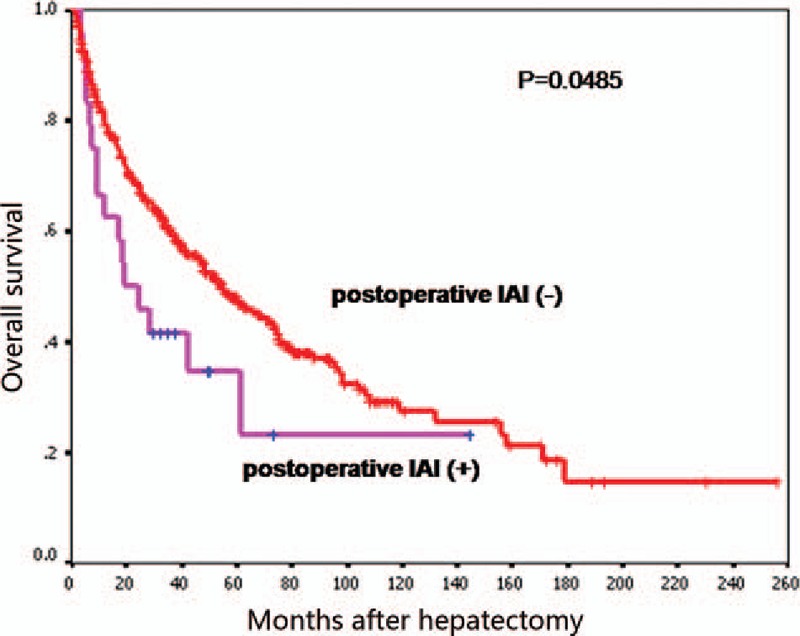
Kaplan–Meier survival curve showing that overall survival of patients with postoperative IAI was shorter than that for those without IAI (*P* < .05). IAI = intra-abdominal infection.

Median overall survival of the 622 patients was 54.1 (95% confidence interval [CI] 44.4–63.7) months and survival at 1, 3, and 5 years was 80%, 60%, and 47%, respectively. Median survival of HCC patients with IAI after hepatectomy was 19.3 (95% CI, 5.8–32.8) months and survival at 1, 3, and 5 years was 63%, 40%, and 34%, respectively. In patients not suffering from IAI, median survival was 55.0 (95% CI, 45.0–65.0) months and survival at 1, 3, and 5 years was 81%, 60%, and 48%, respectively. Survival in the group of patients with IAI was significantly worse than the group of patients without IAI after hepatectomy (*P* = .048; Fig. 1).

### 3.3 Risk factors for postoperative IAI

Risk factors for postoperative IAI included preoperative risk factors (Table 1), tumor-related risk factors (Table 2), and intraoperative risk factors (Table 3). Factors associated with postoperative IAI were: preoperative weight loss, albumin ≤35 g/L, liver cirrhosis, preoperative ascites, operative time >300 minutes, operative blood loss >1000 mL, operative blood transfusion (*P* < .05 for all). Further analyses of these 7 clinicopathological factors by multivariate logistic regression demonstrated weight loss (OR, 3.27; 95% CI, 1.17–9.11; *P* = .024), liver cirrhosis (0.28, 0.12–0.67, .004), and operative time >300 min (3.44, 1.46–8.12, .005) as independent predictors for postoperative IAI (Table 4).

**Table 1:**
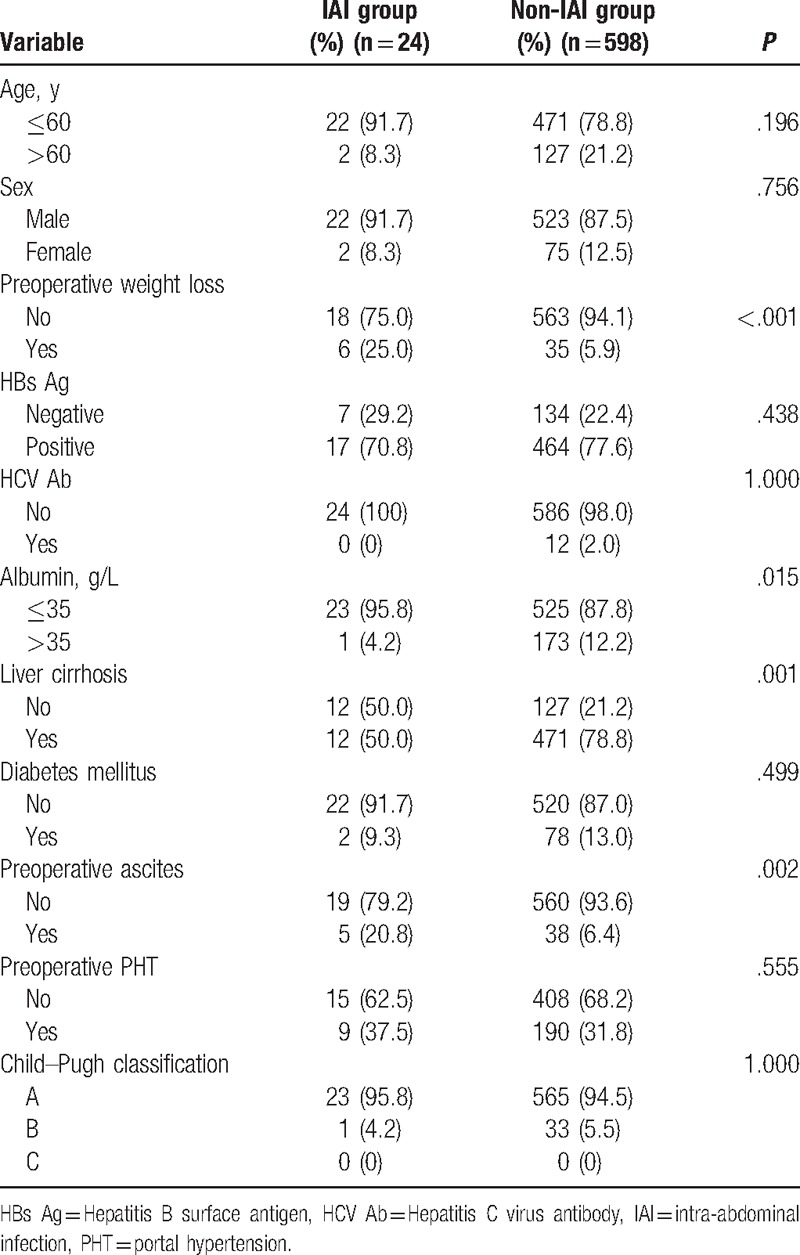
Preoperative risk factors (comparison between IAI group and non-IAI group).

**Table 2:**
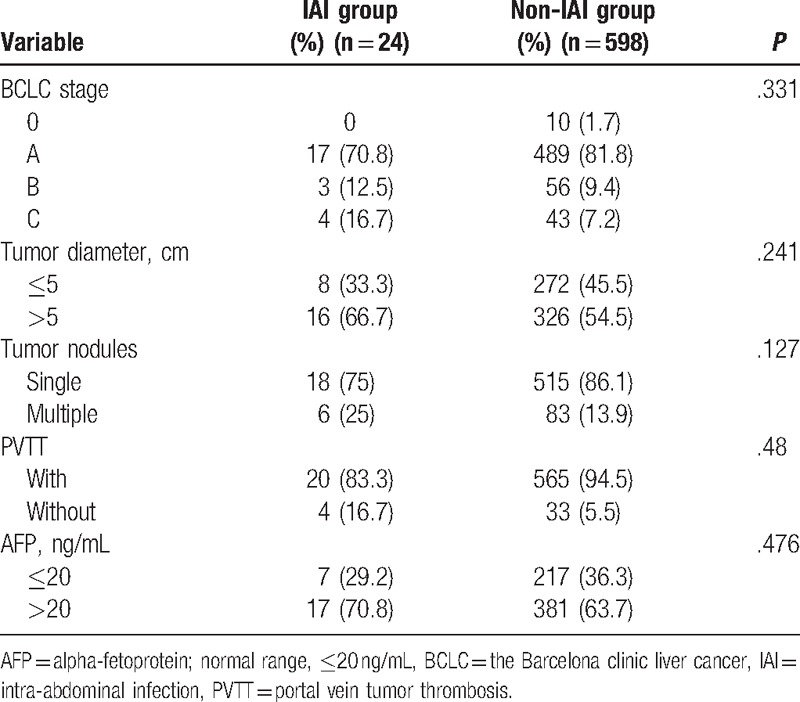
Tumor-related risk factors (comparison between IAI group and non-IAI group).

**Table 3:**
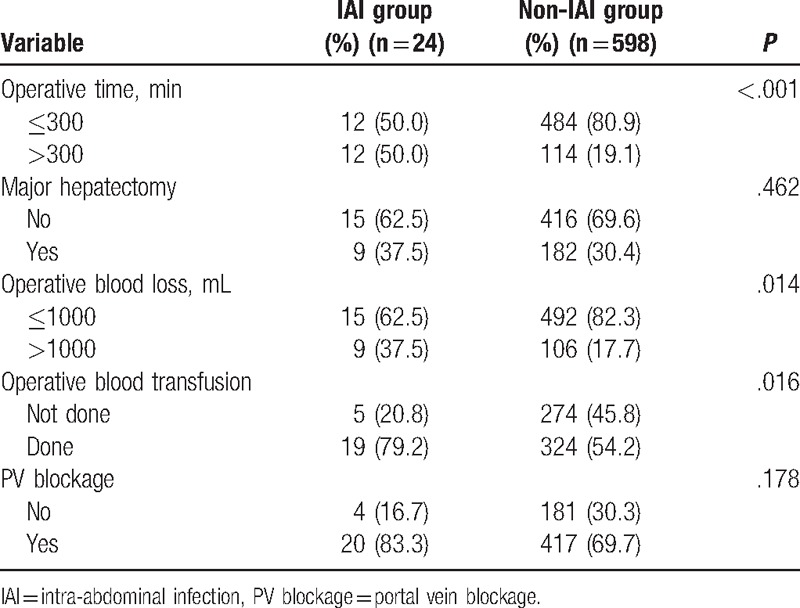
Intraoperative risk factors (comparison between IAI group and non-IAI group).

**Table 4:**
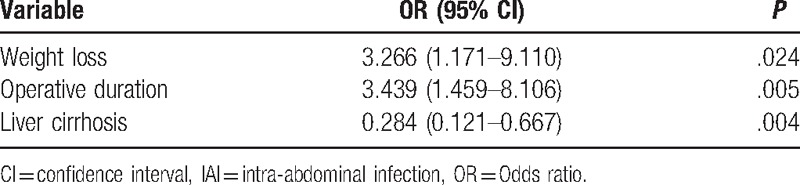
Multivariate stepwise logistic regression analysis of clinical predictors of IAI after hepatectomy.

## 4 Discussion

Our study revealed the negative prognostic significance of postoperative IAI in HCC patients undergoing hepatectomy. A total of 622 patients were evaluated retrospectively and, among them, 24 (3.9%) patients experienced IAI after hepatectomy. Median duration of survival of patients with postoperative IAI was 19.3 months compared with 55 months for those without postoperative IAI. Five-year survival for patients with and without postoperative IAI was 34% and 48%, respectively (*P* = .048). These results demonstrated that postoperative IAI was related significantly to poor long-term survival in HCC patients after hepatectomy. In addition, preoperative weight loss, liver cirrhosis, and operative time >300 minutes were independent predictors for postoperative IAI according to multivariate logistic regression analyses.

The potential mechanisms for survival inferiority of patients with postoperative IAI are beyond the scope of the present study, but might be explained by: amplification of inflammatory and angiogenic responses; suppression of the immune response. Initially, a normal inflammatory response is considered to have critical roles in wound healing. However, magnification of such a response tended to trigger carcinogenesis of normal cells and progression of residual tumor cells.^[30,31]^ Studies have suggested that a postoperative inflammatory reaction results in upregulation of expression of proinflammatory cytokines (interleukin-1, interleukin-6, tumor necrosis factor) and proangiogenic chemokines, thereby inducing and facilitating proliferation of residual tumor cells in their path to recurrence,^[30,32,33]^ which has been confirmed by the close correlation between systemic inflammation and poor survival in various tumor types.^[32,34–36]^ With regard to another aspect of suppression of the immune response, in vitro studies have shown that increased numbers of neutrophils in peripheral blood caused by inflammation inhibit the cytolytic activity of lymphocytes and natural killer cells to tumor cells, and therefore induce immunosuppression.^[33,37]^ Hence, postoperative IAI, by amplification of inflammatory and angiogenic responses as well as inhibition of host immunosurveillance and antitumor immune response, creates a “hospitable” microenvironment in which the survival and expansion of residual tumor cells present in the surgical field, venous blood or occult micrometastases can be supported and promoted in their path to local or distant recurrence.^[38,39]^ Our findings are in close agreement with studies showing that postoperative IAI is a significant risk factor of poor long-term survival in patients with resected HCC.^[40]^

Multivariate analyses revealed that 3 independent predictors for postoperative IAI were significant: preoperative weight loss, liver cirrhosis, and operative time >300 minutes.

Preoperative weight loss was an independent risk factor of postoperative IAI, and could be explained by reduced nutritional reserve and weakened capacity to recover from surgical stress. The inflammatory response exacerbates catabolism within the body. Patients with preoperative weight loss often have a lower nutritional reserve that cannot sustain such exacerbated catabolism. Therefore, patients experiencing preoperative weight loss are more vulnerable to postoperative IAI. As documented previously, an adequate amount and duration of preoperative nutritional support reduces the prevalence of infectious complications significantly.^[41]^ Preoperative weight loss has been associated with adverse postoperative outcomes (including inflammatory complications) in other areas of medicine.^[42–44]^

Interestingly, our present study revealed that rates of postoperative IAI in the non-cirrhotic patients were considerably higher than in the cirrhotic patients (8.63% versus 2.48%, *P* = .001), which appeared to be in contrast with results from previous reports.^[40,45]^

On one hand, such discrepancy might be related to the differences of resected liver volume between non-cirrhotic and cirrhotic patients. In our case series, the mean tumor size in non-cirrhotic and cirrhotic patients were 7.74 ± 3.90 and 6.68 ± 3.90 cm (mean ± SD, *F* = 7.94, *P* = .005), respectively. Namely, patients without cirrhosis featured remarkably larger tumor diameter than those with cirrhosis, which might be partially attributed to the regular monitoring of the liver disease for cirrhotic patients. And this kept in concordance with the results of earlier literatures.^[46,47]^ Grazi et al^[46]^ analyzed a total of 443 HCC patients receiving curative resection over 20 years and found that non-cirrhotic patients presented significantly larger mean tumor diameter (*P* < .01) in comparison with cirrhotic patients; specifically, the maximal tumor diameter in non-cirrhotic and cirrhotic patients were 7.90 ± 4.60 and 4.30 ± 2.10 cm (mean ± SD, *P* < .01), respectively.^[46]^ Considering this, it is reasonable that non-cirrhotic patients in our cohort received resection of larger portions of liver parenchyma. Potentially, greater loss of liver parenchyma would weaken the tolerance for surgical stress. On this occasion, non-cirrhotic patients would carry a more substantial risk of postoperative infectious complications. As documented previously, postoperative infectious complications including wound infection and intra-abdominal collections presented clear preferences to more extensive parenchymal resection.^[48–50]^

However, on the other hand, it must be taken into account that the current series enrolled only 24 patients with postoperative IAI and this inevitably would lead to bias in our study.

Additionally, we found that operative time >300 minutes served as a significant risk factor of postoperative IAI, a result that was in agreement with previous reports.^[51,52]^ Specifically, Cruse and Foord documented that the risk of postoperative infection doubles with each additional hour of surgery.^[53,54]^ Furthermore, the prevalence of postoperative infections increases significantly with duration of the surgical procedure: 6.3% for 1 hour, 12.2% for 1 to 2 hour, and 27.7% for >2 hour.^[51]^ Such results may be because a prolonged operative time carries increased exposure of enterocoelia to the environment and heightened surgical stress to the immune system. Hence, a prolonged operative time is related negatively to the prevalence of postoperative IAI.

With the aim of lowering the prevalence of postoperative IAI, various measures could be applied to diminish the risk factors for postoperative IAI. First, nutritional status should be assessed carefully preoperatively. For those suffering from malnutrition or weight loss, well-managed and intensive preoperative nutritional support should be provided to improve nutritional status and thus promote postoperative recovery. Second, with regard to patients suffering from liver cirrhosis, supportive therapies (e.g., nutritional support) should be applied to improve the physical condition. In particular, for patients with ascites, a combination of pharmacologic and non-pharmacologic therapies is advocated to manage ascites and fluid retention. Restricted salt consumption and diuretics are recommended as first-line therapy.^[55]^ Third, operative time is an unmodifiable factor, so periodic reinjection of antibiotic agents (according to half-life) during the intervention is recommended. For those experiencing an operative time >300 minutes, prolonged or supplementary use of prophylactic antibiotics for the prevention of postoperative IAI might be worthwhile.

Our study had two main limitations: it was a retrospective study, which restricted the comprehensive collection of the data; the number of patients in the IAI group was rather small and bias might remain in our study. For example, predictive values of liver cirrhosis might be conflicting with available data. Hence, further validation by multicenter prospective studies is required.

## 5 Conclusions

The present study suggested that postoperative IAI is closely correlated with poor long-term outcome in HCC patients undergoing curative hepatectomy. Preoperative weight loss, liver cirrhosis, and operative time >300 minutes are independent predictors of postoperative IAI. Therefore, establishing individual strategies such as nutritional support and anti-infectious treatment for patients at high risk might prevent postoperative IAI and further improve the long-term survival of HCC patients.

## Acknowledgments

The authors gratefully acknowledge the statistical guidance and assistance of Professor Xinyuan Tong from Department of Medical Statistics, Medical School of Chinese PLA. They also thank all patients who took part in this clinical research.
